# Reduced Levels and Disrupted Biosynthesis Pathways of Plasma Free Fatty Acids in First-Episode Antipsychotic-Naïve Schizophrenia Patients

**DOI:** 10.3389/fnins.2020.00784

**Published:** 2020-07-29

**Authors:** Xiang Zhou, Tao Long, Gretchen L. Haas, HuaLin Cai, Jeffrey K. Yao

**Affiliations:** ^1^Department of Pharmaceutical Sciences, University of Pittsburgh School of Pharmacy, Pittsburgh, PA, United States; ^2^Medical Research Service and The VISN 4 Mental Illness Research, Education, and Clinical Center, VA Pittsburgh Healthcare System, Pittsburgh, PA, United States; ^3^Department of Psychiatry, University of Pittsburgh School of Medicine, Pittsburgh, PA, United States; ^4^The Department of Pharmacy, The second Xiangya Hospital of Central South University, Changsha, China; ^5^Institute of Clinical Pharmacy, Central South University, Changsha, China

**Keywords:** first-episode schizophrenia, free fatty acids, antipsychotic-naïve, fatty acid pathway, plasma

## Abstract

Membrane phospholipid deficits have been well-documented in schizophrenia (SZ) patients. Free fatty acids (FFAs) partially come from the hydrolysis of membrane phospholipids and serve as the circulating pool of body fatty acids. These FFAs are involved in many important biochemical reactions such as membrane regeneration, oxidation, and prostaglandin production which may have important implications in SZ pathology. Thus, we compared plasma FFA levels and profiles among healthy controls (HCs), affective psychosis (AP) patients, and first-episode antipsychotic-naïve schizophrenia (FEANS) patients. A significant reduction of total FFAs levels was observed in SZ patients. Specifically, significant reductions of 16:0, 18:2n6c, and 20:4n6 levels were detected in FEANS patients but not in APs when compared with levels in HCs. Also, disrupted metabolism of fatty acids especially in saturated and n-6 fatty acid families were observed by comparing correlations between precursor and product fatty acid levels within each fatty acid family. These findings may suggest an increased demand of membrane regeneration, a homeostatic imbalance of fatty acid biosynthesis pathway and a potential indication of increased beta oxidation. Collectively, these findings could help us better understand the lipid metabolism with regard to SZ pathophysiology.

## Introduction

Schizophrenia (SZ) is a devastating neuropsychiatric disorder that affects approximately 20 million people worldwide (GBD 2017 Disease and Injury Incidence and Prevalence Collaborators, 2018). It imposes a massive economic burden on individuals with SZ, their families and the society. Previous studies have linked SZ to widespread structural and functional brain alterations, including multiple neurotransmitter pathway disruptions (Walsh et al., 2008; Kerner, 2009; Karam et al., 2010; Brisch et al., 2014), white matter changes (Kubicki et al., 2005; Lener et al., 2014), and prefrontal-limbic network dysfunctions (Weinberger et al., 1988, 1992). Despite plenty of theories proposed on the pathophysiology of SZ, none of them can fully explain its symptomatology and underlying etiology. Given the diverse biological findings reported in SZ, it is possible that the etiologic heterogeneity of SZ could result from a common pathogenic pathway (or a few pathways) that eventually leads to the clinical syndromes.

As far as we know, currently no literature data illustrating whether direct correlations between levels of plasma free fatty acids (FFAs) and the composition of membrane FAs has been reported. However, there is some evidence from two aspects which suggests indirect associations between them. Firstly, plasma FFAs have long been considered as a pivotal indicator of essential FAs status of humans, reflecting the amount of FA source that can be utilized in membrane phospholipid metabolism (Holman et al., 1979). In support, a study has reported about the FA composition of plasma and erythrocyte membranes in normal individuals (Manku et al., 1983). It shows that most ratios of FAs in plasma are like those in erythrocyte membranes except for 18:2n-6 and 22:4n-6, thought the phenomenon was not discussed. Secondly, *in vitro*, the cellular FA composition is likely to change due to the altered FA levels in serum used to grow established cell lines (Stoll and Spector, 1984). *In vivo*, supplementation of cod fish oil which is rich in omega-3 polyunsaturated fatty acids (PUFA), will result in increased eicosapentanoic and docosahexaenoic acid incorporations into the total phospholipids of plasma, platelets, and erythrocytes in a dose- and time-dependent manner (Von Schacky and Weber, 1985). Moreover, it is well-established that phospholipase A_2_ (PLA_2_) can recognize and cleave FAs from membrane phospholipids. It can bind to the sn-2 acyl bond of phospholipids and catalytically hydrolyze it, releasing arachidonic acid and lysophosphatidic acid into the blood stream (Dennis, 1994). These may suggest that plasma FA and membrane FA composition are dynamically linked.

As a major class of components of myelin, phospholipids play a crucial role in maintaining myelinated neuronal axons in the central nervous system (CNS) and peripheral nervous system (PNS). Studies show that at least 70% of the dry mass of both CNS and PNS myelin is formed by phospholipids (Baumann and Pham-Dinh, 2001; Chrast et al., 2010). Interestingly, previous findings (Gattaz et al., 1990; Horrobin et al., 1991; Pettegrew et al., 1991; Noponen et al., 1993) have indicated that in both CNS and peripheral tissues, SZ patients had an increased breakdown of membrane phospholipids due to PLA_2_ activity. Recent studies have shown that calcium-independent PLA_2_ plays a crucial role in the regulation of phospholipid metabolism and cell membranes functionality in the CNS (St-Gelais et al., 2004; Schaeffer et al., 2005). In addition, increased activities PLA_2_ in SZ patients including drug-naïve patients, drug-free patients and patients with different stages of SZ have been observed (Smesny et al., 2005; Šakić et al., 2016). It is also reported that higher concentrations of PLA_2_ in those patients are positively correlated with the illness duration and episode numbers (Šakić et al., 2016), while other studies suggest significant correlations between PLA_2_ activity and positive symptoms (Gattaz et al., 1990; Ross et al., 1997). The resulting FFAs, which are not bound on cell membranes or proteins, are involved in many important biochemical reactions such as regeneration of membrane phospholipids (Rapoport, 2001; Yehuda et al., 2002), production of prostaglandins (Flower and Blackwell, 1976; Kuehl and Egan, 1980) and generation of adenosine triphosphates via beta-oxidation in the mitochondria. FFAs originate from lipolysis of membrane phospholipids and serve as dynamic lipid pools in the body. Therefore, changes of FFA levels may have significant implications in the SZ pathology. While lots of studies focused on membrane phospholipid abnormalities, no study has investigated the potential role of plasmatic FFAs in SZ pathology. The aim of the current study thus is to test whether levels and biosynthesis of plasma FFAs are altered in SZ patients at early stage of disease development. Levels of FFAs were measured and compared between the HC group and first-episode antipsychotic-naïve schizophrenia (FEANS) group. To test if those potential alterations of FFA profiles were unique features on SZ patients and could be served as potential biomarkers of SZ, the affective psychosis (AP) group including patients with bipolar disorder and major depression was further added to the comparisons of FFAs levels with the HC and FEANS groups. Furthermore, correlations between the levels of different types of fatty acids were explored in distinct groups of patients and controls.

## Materials and Methods

### Clinical Design

Forty SZ antipsychotic-naïve patients were recruited during their first episode of psychosis after they provisionally met diagnostic and statistical manual of mental disorders (DSM-IV) criteria for schizophrenia or schizophreniform disorder based on the Structured Clinical Interview for DSM Disorders. In addition, 52 age-, race-, BMI-, and gender-matched healthy control (HC) subjects and 24 patients with other psychotic disorders (including psychotic bipolar disorder and major depression with psychotic features), herein referred to as AP subjects, were also recruited from the same community. Subjects in AP group were in drug-free status at the time of enrollment. Statistical Manual of Mental Disorders (DSM) version 4 was used to diagnose the patients. To be diagnosed with SZ, two of the following characteristic symptoms need to be met over at least 1 month: delusions, hallucinations, disorganized speech, grossly disorganized or catatonic behavior or negative symptoms (e.g., flattened affect, alogia, amotivation, and avolition). Also, the person should have social or occupational dysfunction or failure to achieve a level of functioning expected for their age and socioeconomic background. Lastly, continuous signs of the disturbance must persist for a minimum of 6 months. No somatic and psychiatric comorbidities were recorded on those SZ patients. None of subjects were taking alcohol or cigarettes for at least 14 days prior to the study or during the study. Also, none of the subjects were on anticoagulants or lipid lowering agents before or during the study. We confirmed that all participants did not have an unbalanced diet, restricted diet or abnormal eating habits. Eating habits between patients and HCs were basically similar. In addition, blood samples were collected after overnight fasting protocol to minimize the diet effect as well. Collected fresh blood was immediately separated for plasma and transferred to the −70°C freezer. Clinical symptoms were evaluated prior to initiation of clinicians’ choice of antipsychotic agents. Scale for the assessment of positive symptoms (SAPS) and scale for the assessment of negative symptoms (SANS) were used with the FEANS and AP group evaluation. The SAPS has 34 items (including four global items) that constitute four subscales measuring hallucinations, delusions, bizarre behavior, and positive formal thought disorder. The SANS has 25 items (including five global items) that constitutes five subscales measuring affective flattening or blunting, alogia, avolition-apathy, anhedonia-asociality, and attention. Each item was scored by clinicians from 0 (no symptom) to 5 (most severe) to evaluate the degree of each symptom. The demographic and clinical characteristics of subjects are shown in Table 1.

**TABLE 1:** Demographic data of recruited subjects.

	**HC**	**AP**	**FEANS**	**Comparisons**		
				**HC vs. AP**	**HC vs. FEANS**	**AP vs. FEANS**
Numbers	52	24	40	NA	NA	NA
Sex^*a*^	31/20	13/11	26/14	<0.001	<0.001	<0.001
Age^1^	26.3 ± 7.4	26.9 ± 9.9	22.7 ± 8.1	ns	ns	ns
BMI^2^	25.2 ± 4.4	25.2 ± 6.9	23.3 ± 5.3	ns	ns	ns
Race^*b*^	34/10/3/4	17/5/1/1	20/16/2/2	<0.001	<0.001	<0.001
SAPS^3^	NA	18.2 ± 14.9	26.0 ± 12.2	NA	NA	0.0265
SANS^4^	NA	37.6 ± 9.5	44.1 ± 11.1	NA	NA	0.0202

*^*a*^Male/Female. Significant differences were observed in Sex ratios as tested by Pearson Chi-square test. ^*b*^Caucasian/African American/Pacific Asian/Others. Significant differences were observed in race ratios by comparing ratios of major race (Caucasian) numbers vs. other races numbers in total, as tested by Pearson Chi-square test. ^1,2^No significant difference was found in age, BMI, and SANS values as tested by analysis of variance among three groups. ^3,4^Significant differences were observed in SAPS and SANS between AP and FEANS group as tested by two sample *t*-test. HC, healthy controls; AP, affective psychosis; FEANS, first-episode antipsychotic-naïve schizophrenia patients; NA, Not Applicable; ns, no significant difference; BMI, body mass index; SAPS, scale for the assessment of positive symptoms; SANS, scale for the assessment of negative symptoms. Note: Age, BMI, and clinical scores are expressed as mean ± standard deviation. In HC group, one subject is missing the information of sex and race. Three subjects in FEANS group and one subject in AP group are missing BMI data due to missing height/weight values.*

### Clinical Ethics

The study was approved by the Institutional Review Boards of the University of Pittsburgh and the VA Pittsburgh Healthcare System. All patients and control subjects were recruited from UPMC Western Psychiatric Hospital in Pittsburgh. Diagnostic assessments and clinical symptom ratings were performed by experienced research clinicians, then reviewed and confirmed by senior diagnosticians during diagnostic conferences attended by research faculty experienced psychiatrists. All subjects including controls were provided written informed consent prior to participation in the research procedures.

### Fatty Acid Methylation and Gas Chromatography Analysis

Plasma FFAs were quantitatively determined by capillary gas chromatography (GC) according to the procedure described by Lepage and Roy (1988). In brief, FFAs were extracted from 50 μl of plasma containing internal standard (Tridecanoic acid) and then converted to methyl esters by acetyl chloride-methanol reagent. After the preparation, 1 μl of the resulting fatty acid methyl esters was injected into GC for analysis. The procedure used for GC analysis has been previously described (Yao et al., 2002; Reddy et al., 2004). Briefly, prepared samples including internal standard were injected into the capillary column, with programmed control of oven temperatures. Each sample was run under a splitless injection mode with helium as the carrier gas (3 mL/minute) and with an inlet pressure of 6.5 psi. All major peaks were eluted within 16 min. Typical chromatography from a HC subject is shown in Supplementary Figure 2. These peaks were then identified and determined by comparing the retention times with those of standard mixtures (Supelco, Inc.). Linear standard curve range of this assay is from 0.25 nmol/mL to 1250 nmol/mL. The Lower Limit of Quantification is 0.05 nmol/mL. Concentrations of each FFA were then calculated by Agilent ChemStation. Each sample was tested in duplicates and quality control samples made from the plasma pool were used to check the differences between batches. Intra-assay percentage of CV was below 5.0% and inter-assay percentage of CV was below 6.5%.

### Data Analysis Packages and Procedures

All tests were done using STATA (v 15.0) software packed. Two-tailed *p*-values were applied to test the fatty acid levels between groups. First, All data were checked for skewness by quantile–quantile plots. Second, for FFA levels comparisons, analysis of variance (ANOVA) with Bonferroni corrections on *post hoc* comparisons were conducted across the HC, AP, and FEANS groups. Third, Pearson correlation coefficients were used to test associations between precursor FAs and product FAs within certain fatty acid pathways. Fourth, Pearson correlation coefficients were used to test associations between FAs and clinical scales.

As there were significant differences between groups in terms of sex and race (Table 1), these variables were used as covariates in the statistical analysis. ANOVA analysis with Bonferroni corrections on *post hoc* comparisons were conducted to test differences between FFA levels across the HC, AP, and FEANS groups. The Bonferroni correction was applied when testing multiple null hypothesis in one data set. Therefore, the critical *p*-value was adjusted to the number of comparisons in a specific ANOVA *post hoc* test. Since FFAs levels among three groups (three comparisons) were compared, the critical *p*-value was set to: 0.05/3 = 0.0167. Pearson correlation coefficients were used to test associations between precursor FAs and product FAs within certain fatty acid pathways. These precursor/product FFAs correlation analyses were run with sex and race as covariates. These correlations were utilized to reflect connections between the precursor and product FFAs. We hypothesize that in SZ patients, a disturbance of FFAs pathways will lead to the loss of tight connections between precursor and product FFAs in comparison to those in control groups. Also, correlations between precursor and product FAs can indirectly implicated the activity of the elongase and desaturase as indicated in Supplementary Figure 1. Therefore, Correlation analysis were conducted among major FAs within each FA family (n-6, n-7, n-9, n-3, and saturated fatty acids) pathway. Specifically, 18:2n6, 18:3n6, 20:3n6, 20:4n6, and 22:4n6 were included in n-6 correlation analyses; 16:1n7t, 16:1n7c, and 18:1n7 were included in n-7 correlation analyses; 18:1n9t and 18:1n9c were included in n-9 correlation analyses; 22:5n3 and 22:6n3 were included in n-3 correlation analyses; 14:0, 15:0, 16:0, 17:0, 18:0, 21:0, 22:0, and 17:1 were included in saturated correlation analyses. Bonferroni corrections were applied to the number of comparisons in the correlation analyses. Precursor/product pairs that were analyzed: 18:2n6/18:3n6, 18:3n6/20:3n6, 20:3n6/20:4n6, 20:4n6/22:4n6, 18:2n6/20:3n6, 18:2n6/20:4n6, 18:2n6/22:4n6, 18:3n6/20:4n6, 20:3n6/22:4n6, and 20:3n6/22:4n6 for the n-6 family (Bonferroni corrected α level = 0.05/10 = 0.005); 16:1n7t/16:1n7c, 16:1n7t/18:1n7, and 16:1n7c/18:1n7 for the n-7 family (Bonferroni corrected α level = 0.05/3 = 0.0167); 18:1n9t/18:1n9c for the n-9 family (α level = 0.05); 22:5n3/22:6n3 for the n-3 family (α level = 0.05); 14:0/16:0, 16:0/18:0, 18:0/22:0, 14:0/18:0, 14:0/22:0, 16:0/22:0, 15:0/17:0, and 17:0/17:1 for the saturated fatty acids (Bonferroni corrected α level = 0.05/8 = 0.0063). For the significant correlations that found between n-3 FFAs levels and clinical scales, the corrected Bonferroni corrected α level = 0.05/4 = 0.0125 (SAPS/22:5n3, SAPS/22:6n3, SANS/22:5n3, and SANS/22:6n3).

## Results

### Comparisons of Plasma Fatty Acids Levels Among All Groups

Significantly lower total levels of plasma FFAs were detected in FEANS subjects than in HC and AP subjects (Table 2). Specifically, the reductions of plasma FFAs were mostly noted in saturated and n-6 fatty acid families (Table 2). Among saturated fatty acids, the 16:0 levels were significantly decreased in the FEANS group as compared with those in the HC and AP groups (Table 2). Among the n-6 family of fatty acids, the 18:2n6c and 20:4n6 levels were significantly lower in the FEANS group as compared with the HC and AP groups (Table 2). On the other hand, no significant differences were observed in each of these FFA levels between HC and AP groups. Among the n-3 family of fatty acids, significantly lower total levels of n-3 FFAs were observed in the FEANS group as compared with the HC group. However, no significant differences in n-3 FFA levels were detected between the FEANS group and the AP group compared to the n-3 FFAs.

**TABLE 2:** Fatty acid concentrations in healthy controls (HCs, *n* = 52), affective psychosis (AP, *n* = 24), and first-episode antipsychotic-naïve schizophrenia patients (FEANS, *n* = 40).

**Fatty acid^*a*^**	**HC**	**AP**	**FEANS**	**Comparisons**			
				**F^*b*^**	**HC vs. AP**	**HC vs. FEANS**	**AP vs. FENAS**
**Saturates**							
14:0	35 ± 16	29 ± 12	34 ± 20	1.065	ns	ns	ns
**15:0**	4.5 ± 1.8	5.9 ± 1.6	3.9 ± 1.9	**9.313**	0.002	ns	<0.0001
**16:0**	357 ± 95	356 ± 82	295 ± 74	**6.889**	ns	0.0007	0.007
17:0	6.6 ± 4.5	6.6 ± 2.7	4.9 ± 1.8	3.001	ns	ns	ns
18:0	109 ± 30	125 ± 28	98 ± 21	**7.917**	ns	ns	0.0004
21:0	5.1 ± 3.6	6.5 ± 2.5	4.5 ± 3.6	2.615	ns	ns	ns
22:0	11.2 ± 4.7	12.6 ± 4.5	9.0 ± 3.2	**6.160**	ns	ns	0.001
**Monoenes**							
**16:1n7t**	5.8 ± 2.4	6.9 ± 2.3	4.5 ± 1.6	**9.963**	ns	ns	<0.0001
**16:1n7c**	22.6 ± 10.7	26.7 ± 11.2	17.1 ± 7.8	**7.577**	ns	ns	0.0003
17:1	9.2 ± 7.0	12.2 ± 4.1	7.8 ± 5.3	**4.053**	ns	ns	0.014
18:1n9t	25 ± 14	27 ± 11	21 ± 12	1.864	ns	ns	ns
18:1n9c	227 ± 73	213 ± 54	181 ± 46	**6.549**	ns	0.001	ns
18:1n7	25 ± 10	28 ± 8	22 ± 8	**4.304**	ns	ns	0.006
**Dienes**							
**18:2n6c**	314 ± 75	322 ± 65	255 ± 62	**10.511**	ns	<0.0001	<0.0001
20:2n6	5.1 ± 3.6	5.1 ± 2.3	3.6 ± 2.4	**3.594**	ns	0.013	ns
**Trienes**							
18:3n6	5.6 ± 2.7	5.3 ± 1.9	4.6 ± 2.1	2.445	ns	ns	ns
20:3n6	5.3 ± 2.9	5.3 ± 2.3	4.3 ± 2.8	1.605	ns	ns	ns
**Tetraenes**							
**20:4n6**	70 ± 17	75 ± 10	60 ± 16	**7.748**	ns	0.004	0.0004
22:4n6	13.4 ± 4.6	15.4 ± 4.1	12.9 ± 4.5	2.527	ns	ns	ns
**Pentanenes**							
22:5n3	5.0 ± 2.6	5.7 ± 2.2	3.9 ± 2.7	**3.869**	ns	ns	0.009
**Hexananes**							
**22:6n3**	26 ± 9	25 ± 7	22 ± 3	**4.584**	ns	0.004	ns
**Totals**							
**Saturates**	529 ± 129	542 ± 118	450 ± 91	**7.012**	ns	0.001	0.002
n-3 fatty acids	31 ± 10	31 ± 8	26 ± 5	**5.756**	ns	0.006	ns
**n-6 fatty acids**	413 ± 90	428 ± 65	340 ± 74	**12.509**	ns	<0.0001	<0.0001
**Grand totals**	1288 ± 297	1315 ± 213	1069 ± 205	**10.738**	ns	<0.0001	0.0003

*^*a*^All concentration values are expressed as mean ± standard deviation. Units: nmol/mL. ^*b*^*F*-value with significant *p* in the analysis of variance (ANOVA) analysis has been shown in bold. Critical *p*-values after Bonferroni Correction: 0.0167.*

### Correlations Within Fatty Acid Families in HC, AP, and FEANS Groups

**FIGURE 1:**
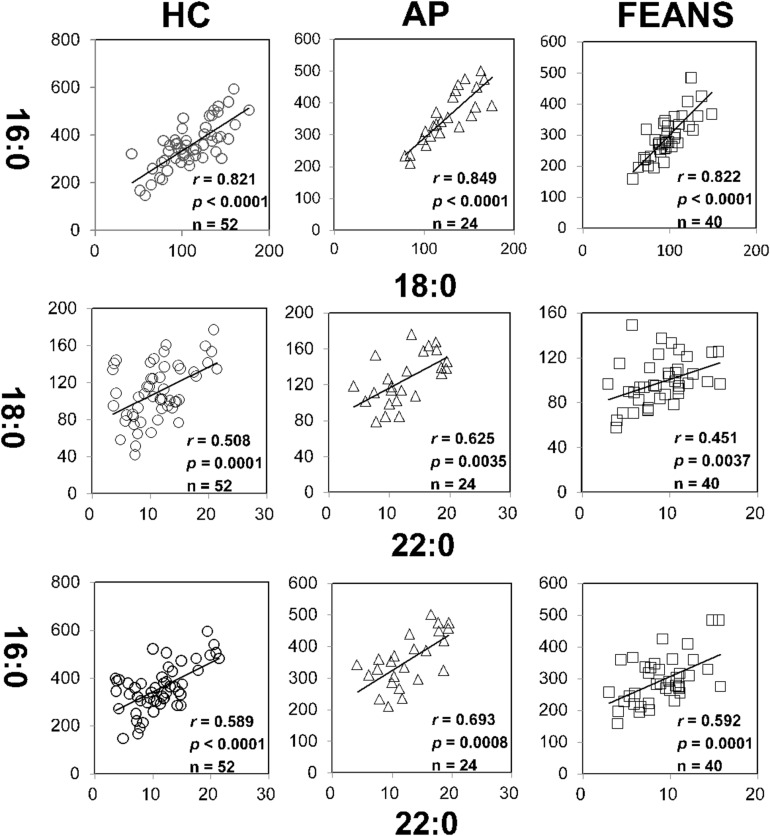
Tight correlations of fatty acids among healthy control (HC) subjects, affective psychosis (AP) patients, and first-episode antipsychotic-naïve schizophrenia patients (FEANS). Critical *p*-value after Bonferroni correction: 0.0063.

There was no significant correlation between any covariate (sex/age) and any FFA index. In the present study, correlations between fatty acids within each fatty acid family were also examined with sex and age as covariates. As expected, there were significant correlations between product and precursor within certain fatty acid families, among the HC, AP, and FEANS groups. Specifically, significant correlations were detected mainly in saturated fatty acids, between 16:0 and 18:0, 16:0 and 22:0, and between 18:0 and 22:0 (Figure 1). For the other families of fatty acids, however, significant correlations were only observed in one or two of the three groups.

**FIGURE 2:**
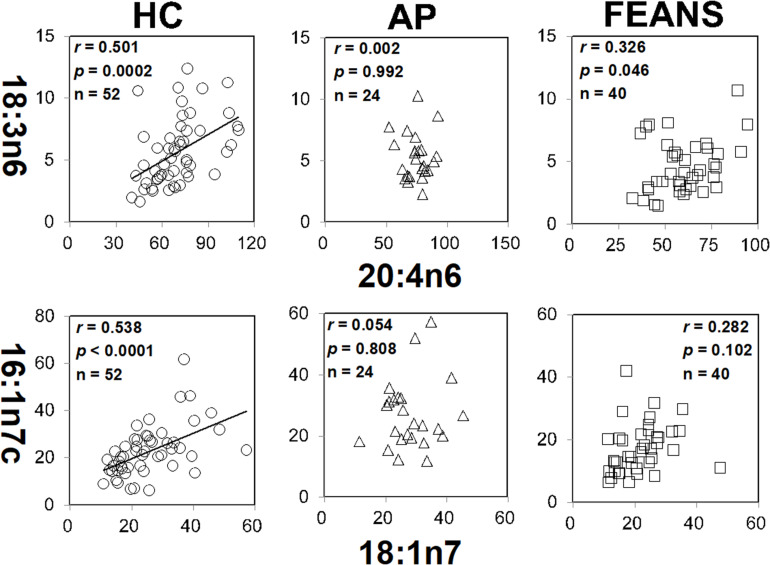
Disrupted correlations of fatty acids both shown in affective psychosis (AP) patients and first-episode antipsychotic-naïve patients (FEANS). Critical *p*-value after Bonferroni correction: for 18:3n6/20:4n6, critical *p* = 0.005; for 16:1n7c/18:1n7, critical *p* = 0.0167.

**FIGURE 3:**
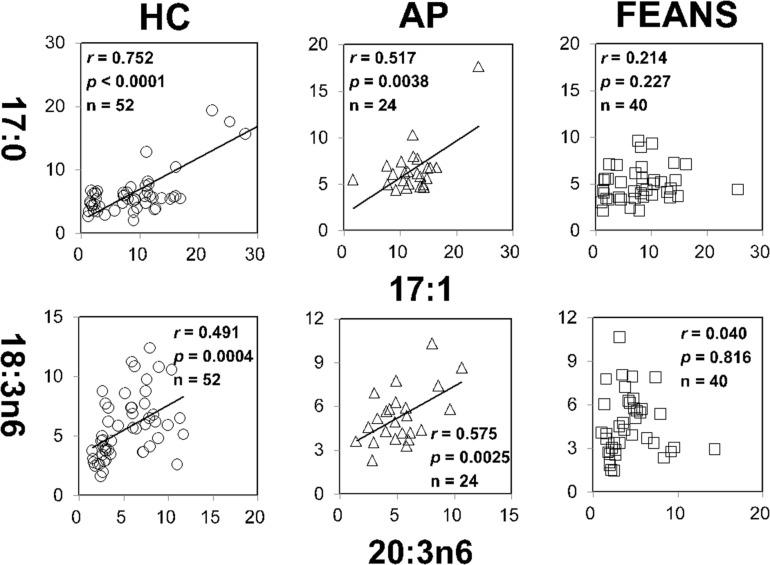
Disrupted correlations of fatty acids only present in first-episode antipsychotic-naïve patients (FEANS). Critical *p*-value after Bonferroni correction: for 17:0/17:1, critical *p* = 0.0063; for 18:3n6/20:4n6, critical *p* = 0.005.

In contrast with the findings mentioned above, significant correlations between 18:3n6 and 20:4n6, as well as between 16:1n7c and 18:1n7, were identified only in the HC group, but not in FEANS or AP groups (Figure 2). Interestingly, significant correlations between 17:0 and 17:1, as well as between 18:3n6 and 20:3n6, were observed both in HC and AP groups, but not in the FEANS group (Figure 3).

### Correlations Between Free Fatty Acid Levels and Clinical Scales in FEANS and AP Groups

**FIGURE 4:**
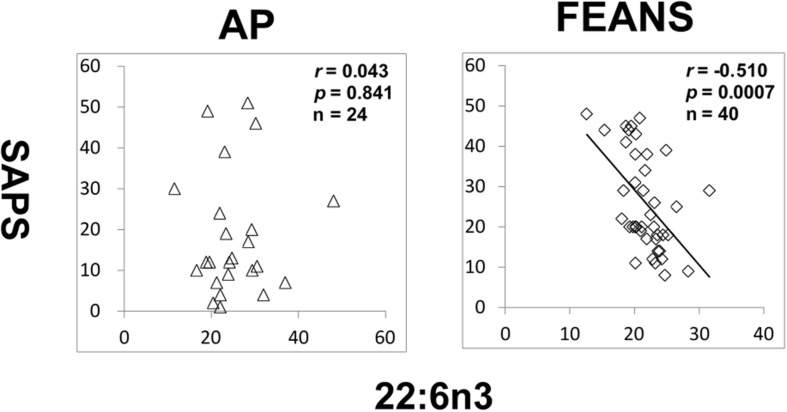
Significant reversed correlations between 22:6n3 and assessment of positive symptoms (SAPS) in first-episode antipsychotic-naïve patients (FEANS). Critical *p*-value after Bonferroni correction: *p* = 0.125.

In FEANS and AP groups, no significant correlation was found between SANS scores and total or any single FFA levels. However, we observed a significant reversed correlation between levels of 22:6n3 and SAPS scores in the FEANS group when comparing with that in the AP group (Figure 4).

## Discussion

In this study, levels of plasma FFAs were determined in SZ patients experiencing their first episode of schizophrenia, patients with a first episode of other (non-schizophrenia) psychotic disorders and HC subjects. Understanding FFA profiles will help us to better comprehend the altered lipid metabolism in SZ. A recent meta-analysis study has revealed that several polyunsaturated FAs levels were decreased in erythrocyte membranes of SZ patients (Van der Kemp et al., 2012). It has also been well-documented that the increased breakdown of membrane phospholipids is mainly due to elevated PLA_2_ activities in SZ patients (Smesny et al., 2005), both in peripheral tissues (Gattaz et al., 1990; Noponen et al., 1993) and CNS (Horrobin et al., 1991; Pettegrew et al., 1991). It has been well-documented that there is an increased breakdown of membrane phospholipids due to elevated PLA_2_ activity in SZ patients, both in peripheral tissues (Gattaz et al., 1990; Noponen et al., 1993) and CNS (Horrobin et al., 1991; Pettegrew et al., 1991). Gattaz and Brunner (1996) proposed that increased PLA_2_ activity in the prefrontal cortex could contribute to membrane phospholipid deficits and eventually lead to hypofrontality in schizophrenia. Several later studies supported this theory which linked membrane phospholipid deficits to myelin dysfunctions (Schmitt et al., 2004; Peters et al., 2012) and prefrontal cortex abnormality (Taha et al., 2013). It is hypothesized that increased lipolysis of membrane lipids would also be associated with the dyslipidemia in SZ patients. Both increased levels of serum triglycerides and decreased levels of serum high density lipoproteins were found in antipsychotic-naïve SZ patients, suggesting the disturbance of lipid profiles could occur during the early course of the disease (Misiak et al., 2017). The FFAs released from membrane phospholipids after hydrolysis by PLA enter into the circulating system. Therefore, an increased breakdown of membrane phospholipids through PLA could potentially lead to higher levels of FFAs as we originally hypothesized. However, instead of observing increased FFAs resulting from increased breakdown of membrane phospholipids, we observed significantly lower levels of plasma FFAs and disrupted linkages in FFA biosynthesis pathways among FEANS patients. More importantly, we did not observe significantly reduced FFAs levels in AP group subjects, suggesting such reductions may be trait features of SZ. Since many important biochemical reactions were involved in those FFAs such as regeneration of membrane phospholipids (Rapoport, 2001; Yehuda et al., 2002), production of prostaglandins (Flower and Blackwell, 1976; Kuehl and Egan, 1980) and generation of adenosine triphosphates via beta-oxidation in the mitochondria, significantly lower levels of plasma FFAs and breakdown of correlations between precursor/product FAs may serve as biomarkers of SZ, which lead to three important implications with regard to SZ’s pathophysiology.

**FIGURE 5:**
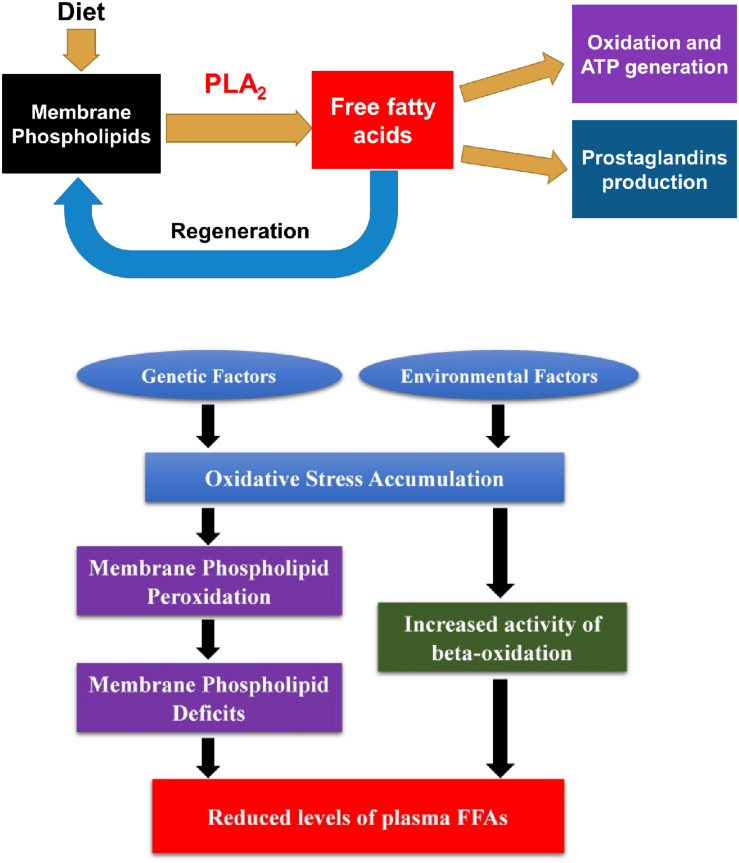
Free fatty acids (FFAs) in lipid metabolism and postulated model of reduced plasma FFA levels and its underlying mechanism in schizophrenia pathophysiology. FFAs come from the lipolysis of membrane phospholipids and serve as an active circulating pool in body. They are involved in many important biochemical reactions including regeneration of membrane phospholipids, beta-oxidation, and production of prostaglandins. In schizophrenia, oxidative stress level was accumulated due to environmental or genetic factors. Consequently, oxidative stress led to membrane phospholipid deficits and increased activity of beta-oxidation of FFAs. Since more FFAs were utilized to regenerate cell membranes and were metabolized through beta-oxidation, levels of plasma FFAs were reduced in SZ patients. PLA_2_, phospholipase A_2_.

First, reduced total FFA levels may imply an increased regeneration of cell membranes and possible alterations in beta-oxidations of fatty acid metabolism at the early course of SZ. While plenty of studies have focused on the PUFA levels in SZ (Van der Kemp et al., 2012), few have discussed other FAs and the total FA levels in SZ (McEvoy et al., 2013; Yang et al., 2017). Recently, a decreased total plasma FFA levels was detected in FEANS patients with limited sample sizes (Zhou et al., 2017, 2018). Unfortunately, the portion of plasma FFAs after the hydrolysis by PLA_2_ from those membrane phospholipids has not yet been explored. In our working hypothesis, reduced levels of plasma FFAs may reflect a depleted pool of those fatty acids resulting from an increased demand of cell membrane regeneration during the early course of SZ development. It is speculated that such demand was due to the membrane phospholipid deficits. As suggested by previous literature, oxidative stress could lead to such deficits through membrane lipid peroxidation in SZ patients (Dietrich-Muszalska and Kontek, 2010; Bitanihirwe and Woo, 2011; Boskovic et al., 2011). Notably, while there is a general trend of decrease among all families of FFAs, we found that n-6 and saturated family FFA levels in FEANS group are significantly lower than those in HC and AP groups. This finding implies that these two fatty acid families are most vulnerable to oxidative stress at the early stage of SZ. As the body cannot synthesize n-6 fatty acids by itself, it might be interesting to discuss the potential dietary effects on these fatty acids here. When the fats get absorbed through digestion system, they will breakdown and then converted to triglycerides and lipoproteins, incorporated into membrane phospholipids and further be broken down into FFAs. Our previous study showed that when comparing membrane-bound fatty acid levels in red blood cell samples, no significant difference in either saturated or monounsaturated fatty acids was observed between FEANS and control groups (Yao et al., 2002). This finding suggested that dietary effects might not have a significant impact on membrane phospholipid levels. In the present study, there was no evidence of metabolic dysfunction observed in these FEANS patients. At the meantime, FEANS subjects’ BMI data were comparable with those in controls (Table 1). Therefore, it’s not likely that the dietary effects played a significant role here. Furthermore, previous studies showed that several fatty acid beta-oxidation enzymes were significantly increased in the brains of SZ patients, which implied enhanced beta-oxidation in FEANS patients (Prabakaran et al., 2004). Up-regulated beta-oxidation in peripheral tissues of SZ patient has also been suggested by comparing product-to-precursor ratios of serum FFAs (Yang et al., 2017). The decreased FFAs levels thus may result from a depleted source in membrane phospholipids as lipids metabolism was shifted toward beta-oxidation in the cytosol. Oxidative stress might play a role in promoting lipid beta-oxidation here, although the impact on FFA metabolism by oxidative stress is still unclear and the mechanism by which it caused the up-regulation of the beta-oxidation remains to be determined. In summary, it is speculated that the reduction of plasma FFA levels may be due to the increased regeneration of membrane phospholipids and hyperactivity of beta-oxidation in SZ pathology. A postulated model is proposed below to explain the reductions of FFA levels (Figure 5) in SZ patients. In brief, the extent of oxidative stress was accumulated in SZ patients due to environmental or genetic factors. The increased oxidative stress levels led to membrane phospholipid deficits, and thus, more FFAs were taken up for regenerating cell membranes in SZ patients. Also, increased oxidative stress levels resulted in an increased activity of beta-oxidation on FFAs, and thus more FFAs were metabolized through this reaction in SZ patients. As a result, reduced levels of total plasma FFAs were observed in the FEANS group when compared with the HC and the AP groups.

Second, our findings reflect a homeostatic imbalance of fatty acid biosynthesis at the early stage of SZ. Most FFAs can be synthesized by elongases and desaturases, and then go through beta-oxidation to generate energy (Supplementary Figure 2). Although some of precursor to product correlations persisted across disease and normal status, some appeared to be lost in the FEANS group. Our results suggest that the potential for steady formation of fatty acids is altered early in the course of illness. While alterations were also observed in the saturated fatty acid family, it appears that the n-6 fatty acid family is the one that was most altered in FEANS subjects but not in the AP subjects. This may suggest that the n-6 fatty acid biosynthesis is more disrupted than other fatty acid families at the early course of the SZ while not in the other psychiatric disorders. Although there was no difference of 22:6n3 between FEANS and AP groups (Table 2), 22:6n3 levels were negatively associated with SAPS scores only in FEANS and this very correlation is absent in AP group (Figure 4). N-6 PUFA (especially arachidonic acid and palmitic acid) can be viewed as proinflammatory molecules, whereas n-3 PUFA (especially eicosapentaenoic acid and docosahexaenoic acid) can be viewed as anti-inflammatory molecules (Sears and Perry, 2015). More disrupted n-6 fatty acid biosynthesis may be considered as a trait-like marker reflecting a severer inflammatory status of SZ patients. However, n-3 PUFA such as 22:6n3 levels could preferably serve as a state-like marker in SZ, which is linked to the symptomatology. However, the exact mechanisms of such alteration on n-6 family FFAs are still unknown. In summary, our findings suggest a disrupted biosynthesis of fatty acids, especially on n-6 fatty acids in the early stage of SZ.

Third, a significantly reduced n-6 FAs may be attributed to the blunted niacin-induced skin flushing in a subgroup of SZ patients, which might be clinically useful in the diagnosis of schizophrenia (Morrow et al., 1989, 1992; Ward et al., 1998; Puri et al., 2001; Benyó et al., 2005; Maciejewski-Lenoir et al., 2006; Tang et al., 2006; Lai et al., 2007; Messamore and Yao, 2016). While the cause of such blunted skin flushing in SZ is still unclear, our findings of a significant decreased arachidonic acid (20:4n6) as well as its precursors in FEANS patients may offer a possible explanation to the underlying mechanism that caused the blunted niacin-induced response in SZ. It is speculated that reduced levels of 18:2n6 and 20:4n6 may reflect a decreased pool for subsequent production of prostaglandin vasodilators (PGD_2_ and PGF_2_), which could lead to the blunted niacin-induced flushing response in those SZ patients (Messamore et al., 2010).

## Limitations

The present study of FFAs was mainly focused on FEANS patients, while antipsychotic treatment effects on those FFAs might also be an interesting research target. This is due to the high prevalence of metabolic syndrome among antipsychotic-treated SZ patients, especially among those treated by second-generation antipsychotics (Casey et al., 2004; Wu et al., 2006). Although promising clinical data showed the metabolic improvement of SZ patient by n-3 FAs supplementation in a randomized placebo-controlled trial (Xu et al., 2019), no literature had reported the possible impact of antipsychotics on FFA profiles in terms of metabolic syndrome. A previous preliminary study was conducted by our group to demonstrate a significant increase of total levels of FFAs after risperidone (a second-generation antipsychotic) treatment when compared to baseline FEANS patients (Zhou et al., 2017, 2018). Future in-depth study will be conducted to investigate the effect of antipsychotics on lipid profiles among SZ patients.

Based on our findings, significantly decreased levels of FFAs within n-6 family were observed in the FEANS group, which may imply a possible link to the blunted niacin-induced skin flushing in a subgroup of SZ patients. However, niacin response data in this clinical group was not available to validate the potential relationship between n-6 FFAs and niacin-induced skin flushing responses. Further studies are required to correlate plasma n-6 FFAs, especially arachidonic acid, with niacin response data. Also, since only a subgroup of SZ patients are presenting such blunted skin flushing, it would be interesting to see if there is a significant difference between a positive-flushing group and a minimal/non-flushing group in terms of arachidonic acid levels.

Most FFAs are synthesized by elongases and desaturases from substrate FFAs, and then go through beta-oxidation to generate energy. However, this study did not measure the activity of elongase and desaturase in those clinical subjects. Instead, comparisons of correlations between precursor and product FFAs among FEANS and control subjects were used to indirectly reflect a possible disruption within metabolic pathways.

Last of all, subjects in the AP group were in drug-free status at the time of enrollment. Patients in this group were recruited after the relapse without at least 30 days of treatment. Therefore, we cannot completely tease out the medication effect on the AP groups in terms of FFA levels. Moreover, low sample size and a lack of information regarding smoking status also became the limitations of the study.

## Conclusion

Significantly reduced plasma FFAs levels were observed for FEANS patients, especially in 16:0, 18:2n6c, 20:4n6, and total FFA levels, when compared with those levels in the (AP and HC) control groups. Also, disrupted correlations of fatty acids within saturated and n-6 fatty acid families were observed. These findings suggested an increased demand of membrane regeneration, a homeostatic imbalance of the fatty acid biosynthesis pathway, and a potential increase in beta oxidations.

## Data Availability Statement

The raw data supporting the conclusions of this article will be made available by the authors, without undue reservation, to any qualified researcher.

## Ethics Statement

All participants provided written informed consent prior to participation in any research procedures. The study was approved by the Institutional Review Boards of the University of Pittsburgh and the VA Pittsburgh Healthcare System.

## Author Contributions

XZ and JY designed the research. XZ and TL performed the research. XZ, TL, GH, and HC wrote the manuscript. All authors contributed to the article and approved the submitted version.

## Conflict of Interest

The authors declare that the research was conducted in the absence of any commercial or financial relationships that could be construed as a potential conflict of interest.
