# Supplementary figures and images for: Reduced Levels and Disrupted Biosynthesis Pathways of Plasma Free Fatty Acids in First-Episode Antipsychotic-Naïve Schizophrenia Patients
Source: Front Neurosci. 2020 Jul 29;14:784. doi: 10.3389/fnins.2020.00784 (PMC7403507; doi:10.3389/fnins.2020.00784)

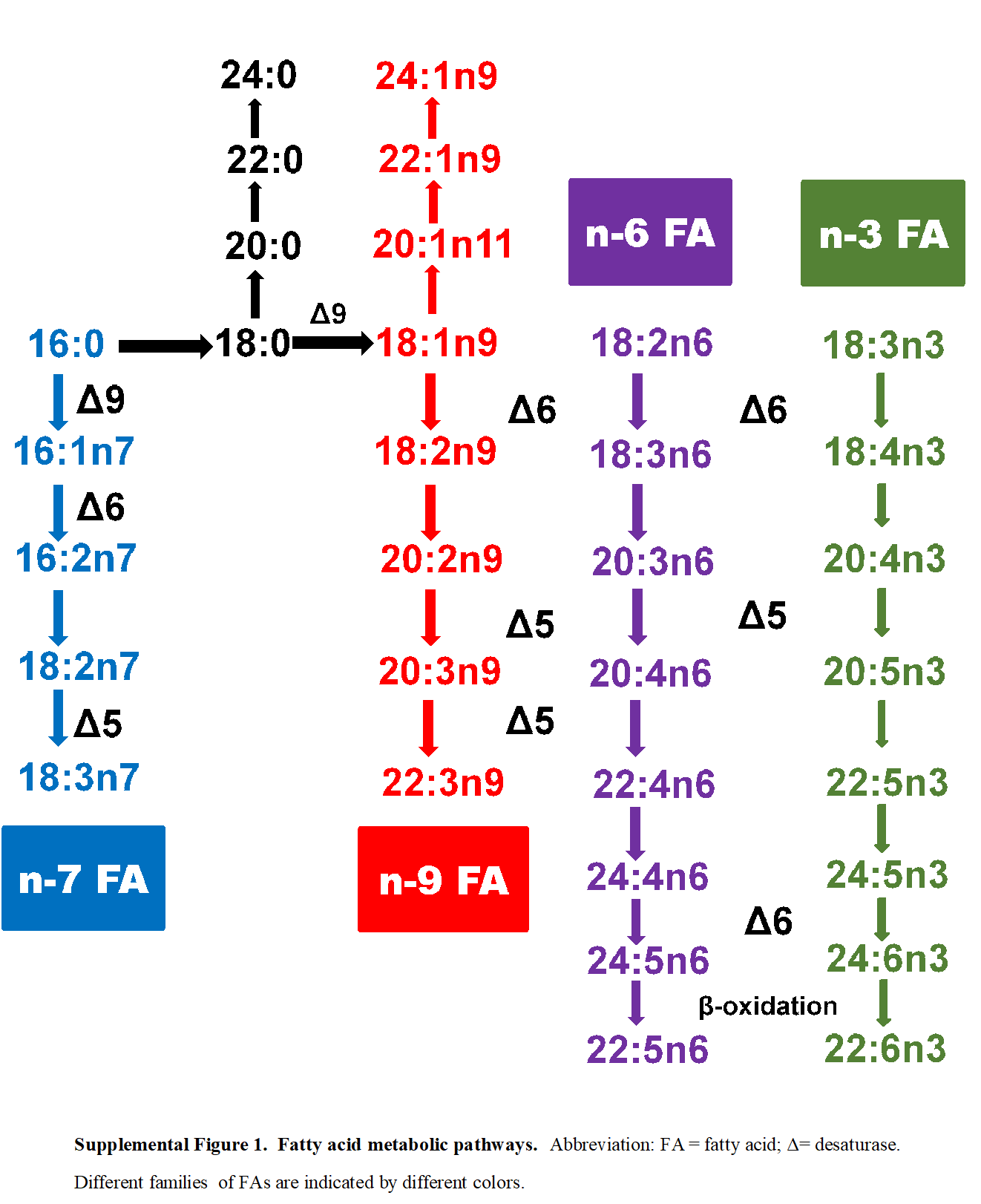

Supplement: Supplementary file 1 [file Image_1.TIF]

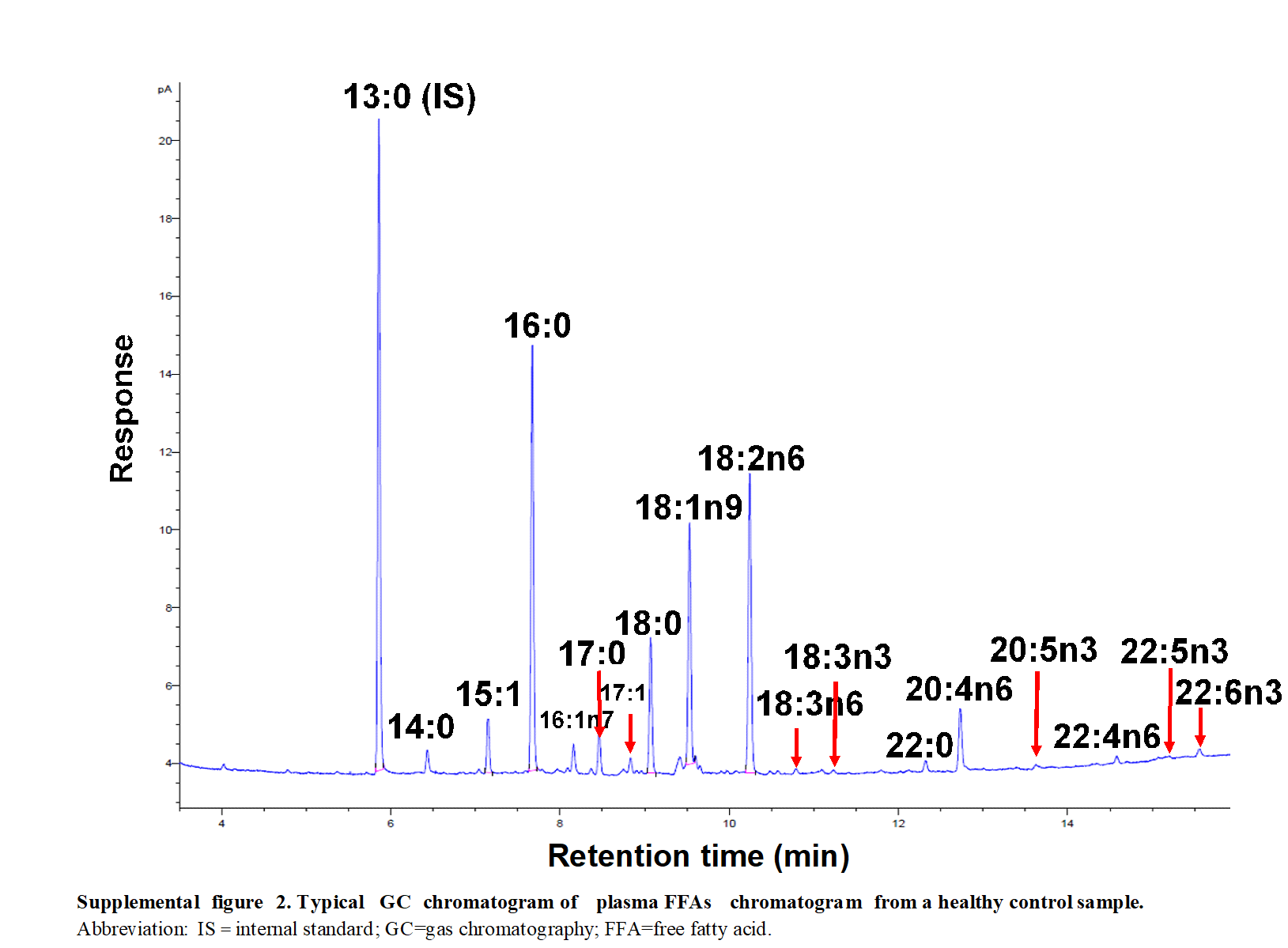

Supplement: Supplementary file 2 [file Image_2.TIF]
